# Achievement of Tolerance Induction to Prevent Acute Graft-vs.-Host Disease

**DOI:** 10.3389/fimmu.2019.00309

**Published:** 2019-03-06

**Authors:** Govindarajan Thangavelu, Bruce R. Blazar

**Affiliations:** Division of Blood and Marrow Transplantation, Department of Pediatrics, Masonic Cancer Center, University of Minnesota, Minneapolis, MN, United States

**Keywords:** graft-vs.-host disease, immune tolerance, alpha-1 antitrypsin, allogeneic hematopoietic stem cell transplantation, T regulatory cells

## Abstract

Acute graft-vs.-host disease (GVHD) limits the efficacy of allogeneic hematopoietic stem cell transplantation (allo-HSCT), a main therapy to treat various hematological disorders. Despite rapid progress in understanding GVHD pathogenesis, broad immunosuppressive agents are most often used to prevent and remain the first line of therapy to treat GVHD. Strategies enhancing immune tolerance in allo-HSCT would permit reductions in immunosuppressant use and their associated undesirable side effects. In this review, we discuss the mechanisms responsible for GVHD and advancement in strategies to achieve immune balance and tolerance thereby avoiding GVHD and its complications.

## Introduction

Immunological tolerance is a self-regulatory mechanism of the immune system to protect the host from a wide variety of foreign antigens without causing immunopathology such as autoimmunity (1, 2). The mechanisms of immunological tolerance can be divided into central and peripheral tolerance. Central tolerance involves the clonal deletion of self-reactive lymphocytes in the primary lymphoid organs, namely the thymus and bone marrow. Despite its high efficiency, central tolerance often is incomplete due to the escape of self-reactive lymphocytes into the periphery. Hence, there is need of an additional layer of tolerance in the periphery to suppress self-reactive lymphocytes. Peripheral tolerance mechanisms consist of deletion, anergy, ignorance and immune regulation (2, 3).

Although significant progress has been made toward immunological tolerance induction in experimental animal models, translation to the clinic for allogeneic hematopoietic stem cell transplantation (allo-HSCT) remains challenging. One manifestation of tolerance induction failure in allo-HSCT is graft-vs.-host disease (GVHD), a life-threatening complication due to donor T cell recognition of host alloantigens. During GVHD, conditioning regimen induced tissue injury drives proinflammatory processes that support the priming of donor anti-host alloreactive T cells via T cell receptor (TCR) engagement, co-stimulation and cytokine signaling. These inflammatory events are counteracted by anti-inflammatory processes often augmented by proinflammatory cytokines; however, for those that develop GVHD, it is clear that anti-inflammatory compensatory mechanisms are overwhelmed and hence unable to control T-cell activation, differentiation and expansion (4, 5). This review will focus on acute GVHD pathogenic and tolerance mechanisms including as available clinical trial results and conclude with the concept of tissue tolerance. Since GVHD acquisition is a sign of failed tolerance induction, we will not discuss GVHD therapy.

## Overview of Alloreactive T-cell Activation, Amplification and Migration

In allo-HSCT, donor CD4+ and CD8+ T cells can receive TCR signals engagement of peptide- major histocompatibility complex (MHC) (termed signal 1) that occurs as a result of major or minor histocompatibility antigen disparities between donor and host. Studies from mouse models revealed that donor CD4+ T cells play a central role in GVHD induction by exhibiting cytolytic activity, producing effector cytokines and helping donor CD8+ T cells to proliferate via IL-2 production (6).

Upon alloantigen activation, CD4+ T cells differentiate into T helper (Th) cell subsets including, most relevant to this review, Th1 (secreting IL-2, IFN-γ), Th2 (secreting IL-4, IL-5, IL-10, IL-13) and Th17 (secreting IL-17A, IL-17F, IL-21, IL-22, TNF) (6). Our group and others have previously provided evidence against the assumption that GVHD is strictly a Th1 driven process (7–9). In our previous study, deletion of IFN-γ in donor inoculum accelerated GVHD lethality, while deletion of IL-4 resulted in reduced GVHD lethality (7). In other studies, Th2 and Th17 subsets were shown to contribute to GVHD severity with different GVHD target organs (8–10). Recently a subset of CD4+ T cells was found to produce GM-CSF that was linked to the support of GVHD pathology by licensing myeloid cells to produce IL-1 and reactive oxygen species (11).

Similar to CD4+ T cells, CD8+ T cells have been implicated as contributing to GVHD in both major and minor histocompatibility models, the former typically in conjunction with CD4+ T cells and contributing to tissue injury, whereas in the latter, CD8+ T cells alone can be sufficient to cause GVHD (12–15). Similar to CD4+ T cells, CD8+ T cells can also differentiate to cytokine producing subsets including Tc1, Tc2, and Tc17 subsets. These CD8+ subsets possess variable capacities to induce acute or chronic GVHD (cGVHD) (16, 17).

A second or co-stimulatory signal (termed signal 2) then is required for full CD4+ and CD8+ T cell activation, expansion, differentiation, survival, and metabolic fitness. Previous studies (18–22) delineated the role of co-stimulatory molecules including CD28 (18), ICOS (CD278) (19), CD40L (CD154), OX40 (CD134) (20), and 4-1BB (CD137) (21). Co-inhibitory molecules can counterbalance co-stimulatory molecules. Cytotoxic T-lymphocyte–associated antigen 4 (CTLA-4; CD152) (23), programmed death−1 (PD-1; CD279) and its ligand (PD-L1; CD274) (24, 25), B and T lymphocyte attenuator (CD272) (26), and B7-H3 (CD276) (27) have been shown to attenuate GVHD lethality. A third signal provided by inflammatory cytokines such as IL-12 or type 1 interferon is required for optimal CD8+ T cell function (28, 29).

An amplifying component of the immune response is ascribed to conditioning-related tissue damage releases damage-associated molecular pattern (DAMPs) and pathogen-associated molecular pattern (PAMPs) molecules (5). These molecules initiate immune responses during the early phases of GVHD and also provide a source of inflammatory cytokines that drive T cell responses. The role of DAMPs in accelerating GVHD lethality was illustrated by the binding of extracellular ATP and subsequent signaling of the purinergic P2X7 and P2Y2 receptors in host antigen presenting cells (APCs) bolstering donor T cell priming and alloreactive responses (30, 31). Conversely, ecto-nucleotidases such as CD39 and CD73, which regulate extra-cellular ATP levels, play suppressive roles in controlling GVHD (32–34). Other DAMPs such as uric acid, IL-33, heparan sulfate, high-mobility group box 1 protein, sialic acid–binding immunoglobulin-type lectins, mitochondrial components, and biglycans fuel GVHD responses (5).

The role of bacterial components in activating APCs and promoting GVHD via PAMPs is well established (35). For example, lipopolysaccharides are toll-like receptor 4 ligand and are implicated in marshaling innate immunity reactions, NF-κB activation, and transcription of pro-inflammatory cytokines genes (35). DAMPs and PAMPs not only contribute to GVHD initiation but also may augment later allogeneic T cell activation, differentiation, and expansion. Priming of allo-reactive donor T cells most often occurs in secondary lymphoid organs through interaction of the TCR with allo-peptide and MHC antigens expressed on host (termed direct allorecognition) or less often, on donor (termed indirect allorecognition) APCs. Both hematopoietic cells and non-hematopoietic cells are involved in alloantigen presentation that promotes and amplifies GVHD responses (36, 37). Recently, neutrophils have also been shown to exacerbate GVHD lethality by releasing reactive oxygen species in the gastrointestinal (GI) tract and surprisingly up-regulating MHC class II antigens (38, 39).

Chemokines guiding the migration of T cells toward GVHD target organs (40) wherein activated T cells mediate targeted tissue cell death via FAS ligand, perforin/granzymes, and releasing pro-inflammatory mediators mainly tumor necrosis factor (TNF-α), interferon (IFN-γ) (5, 41, 42). Other cytokines such as IL-7, IL-15, and IL-6 directly or indirectly support the expansion or activation of the innate and adaptive immune system and have been implicated in exacerbating GVHD lethality (43, 44). To achieve long term tolerance in allo-HSCT settings, strategies to control T cell activation, differentiation, expansion, and homing are critical to allow anti-inflammatory and central and peripheral regulatory events to be dominant over pro-inflammatory mechanisms. The following sections discuss approaches to blunt the distinct stages of GVHD induction (Table 1).

**Table 1:** Approaches to blunt the distinct stages of GVHD induction.

**Strategies (agent or cell)**	**Mechanism of action**	**Predominant clinical indication**	**References**
**REDUCING DONOR ANTI-HOST ALLOREACTIVE T CELLS**			
Anti-thymocyte globulin	Depletion of donor T cells	Prophylaxis and therapeutics	(45, 46)
Alemtuzumab	Depletion of CD52+ mature lymphocytes	Prophylaxis and therapeutics	(47–49)
Post-transplant cyclophosphamide	Depletion of rapidly proliferating alloreactive donorT cells	Prophylaxis	(50–54)
Exvivo depletion of CD45+ naïve T cells	Depletion of naive T cells	Depletion of naive T cells	(55)
**BLUNTING TCR SIGNALS (Standard approaches usually in combinations)**			
Tacrolimus and Cyclosporine	Calcineurin inhibitors; blocks T cell proliferation and IL-2 transcription	Prophylaxis	(56, 57)
Methotrexate	Folate antagonist; inhibits T cell proliferation	Prophylaxis	(56, 58, 59)
Mycophenolate mofetil	Blocks *de novo* synthesis of purine metabolism; inhibits T cell proliferation	Prophylaxis	(56, 58, 59)
Sirolimus	mTOR inhibitors; block T cell activation	Prophylaxis	(56, 60, 61)
**INHIBITING CO-STIMULATORY SIGNALS**			
CTLA-4 Ig	Inhibits CD28 mediated T cell activation	Prophylaxis	(62)
**IMPAIRING ACTIVATING AND INFLAMMATORY CYTOKINE SIGNALS DRIVEN GVHD INJURY**			
Ruxolitinib, Pacritinib	JAK inhibitors; Block T cell activation, cytokine production, and proliferation	Therapeutics	(63–65)
Alpha-1-antitrypsin (AAT)	Reduces pro-inflammatory cytokine secretion, expands Treg numbers, Inhibits neutophil elastase, decreases CD8+ effector memory cells	Therapeutics	(66–68)
**REGULATING HISTONE DEACETYLASE**			
Histone deacetylase inhibitors (vorinostat)	Reduce pro-inflammatory cytokine secretion, increase Treg numbers, modulate the function of APCs, upregulate IDO expression in DCs	Prophylaxis	(69–71)
**BLOCKING T CELL CHEMOKINE RECEPTOR DIRECTED MIGRATION INTO GVHD ORGANS**			
CCR5 inhibitor (Maraviroc)	Prevents T cell infiltration into GVHD tissues	Prophylaxis	(72, 73)
α4β7 (Natalizumab, Vedolizumab)	Prevents T cell infiltration into intestines	Prophylaxis	(72, 73)
**CELLULAR THERAPY**			
Mixed hematopoietic chimerism	Promotes immune tolerance	Prophylaxis	(74–76)
nTregs	Promotes immune tolerance	Prophylaxis and Therapeutics	(77–79)
iTregs	Promotes immune tolerance	Prophylaxis	(80–82)
Tr1	Promotes immune tolerance	Prophylaxis	(83–85)
MSCs	Immunomodultaor, Tissue repair	Therapuetics	(86, 87)

## Reducing Donor Anti-host Alloreactive T Cell Burden

### *In vitro* or *in vivo* T Cell Depletion

In allo-HSCT, the cellular composition of the graft includes hematopoietic stem cells (HSCs) and a wide variety of cells, which influence engraftment. HSCs restore hematopoietic function, whereas other cell types such as mature T cells promote engraftment by inhibiting graft rejection mediated by recipient immune responses. Although T cells play a central role in the pathogenesis of GVHD, depletion of T cells increases the risk of infection and also of leukemia relapse (88, 89). Donor T cell depletion may be accomplished by *in vitro* or *in vivo* strategies. Pan-T cell depletion of the donor grafts can be highly effective but is associated with increased susceptibility to infections and malignancy recurrence due to the relatively long period of time required to reconstitute the immune system (90). *In vivo* administration of anti-T cell globulin (45, 46) or anti-CD52 mAb, CAMPATH-1 (47–49), reduce the donor T cell burden, while resulting in a state of T cell deficiency.

T cells are broadly classified as naïve vs. antigen experienced memory T cells (TM) (91). Stage of T cell differentiation is a critical factor in determining the capacity of T cells to induce GVHD. For instance, unlike naïve T cells, alloreactive effector and central TM cells failed to induce GVHD in pre-clinical models (92–94). The reduced ability of TM cells to induce GVHD is attributed to their reduced survival, expansion and alloreactivity (95). In a first-in-human trial, depletion of CD45RA+ naïve T cells from peripheral blood stem cells did not reduce the incidence of GVHD (55). Nonetheless, all patients with GVHD uniformly responded to corticosteroids (55). A recent clinical trial (NCT01523223) used a final infusate of highly purified (>94%) CD8+ TM cells to treat relapse after allo-HSCT patients (96). Consistent with the results of pre-clinical models, CD8+ TM infusions are associated with low incidence of GVHD (1 of 15 patients, grade II liver GVHD). Altogether, strategies employing T cell grafts depleted of Tnaive cells may facilitate immune tolerance in allo-HSCT settings by hampering pro-inflammatory responses.

### *In vivo* Post-transplant Cyclophosphamide Induced Alloreactive T Cell Depletion

In a recent approach, cyclophosphamide (Cy) that has both anti-neoplastic and immune modulatory effects, has been used to deplete alloreactive donor T cells and thereby prevent GVHD (50–52). Post-transplant cyclophosphamide (PTCy), typically given for 2 consecutive daily doses between days 3–5 post-transplant in combination with calcineurin inhibitors (CNI) and mycophenolate mofetil (53, 97, 98) or as a single agent (99, 100). Cy, a cytotoxic alkylating agent, specifically targets rapidly proliferating alloreactive T cells because of their impaired ability to replicate their damaged DNA (100–102). On the other hand, Tregs are relatively resistant to PTCy through increased expression of aldehyde dehydrogenase enzyme (103), which converts active to inactive Cy metabolites. The expansion and induction of Tregs promotes peripheral tolerance by suppressing remaining allo-reactive T cells and also hastens immune reconstitution. The final step for achieving long-term tolerance induced by PTCy is mediated by the later stage intrathymic deletion of immature alloreactive donor T cells. In clinical trials, PTCy reduced GVHD in both HLA-matched and partially HLA-mismatched allo-HSCT patients (53, 54). There are multiple ongoing clinical trials (NCT01028716, NCT01349101, NCT01860170, NCT02053545, NCT02065154, NCT02167958, NCT02169791) to investigate the effects of PTCy in conjunction with other agents to prevent GVHD. Overall results of clinical trials have shown a reduction in acute GVHD with a pronounced reduction in cGVHD albeit with organ toxicity, carcinogenicity and increased rates of infections.

## Blunting TCR Signals

Standard pharmacological regimens to prevent acute GVHD involve calcineurin inhibitors (CNI), mammalian target of rapamycin (mTOR) inhibitors, and anti-metabolites (5, 56). Calcineurin inhibitors such as tacrolimus or cyclosporine inhibit IL-2 production and subsequently clonal expansion of activated T cells (57). Sirolimus, a lipophilic macrocytic lactone, which binds to FKBP12, and inhibits the mTOR kinase activity, reducing cytokine responses and regulating cell proliferation, survival and metabolism by integrating information from environmental cues including stress signals such as nutrient deprivation (60). TCR, IL-2, CD28, sphingosine-1-phosphate receptor and leptin signals up-regulate the mTORC1 complex. Unlike CNI, sirolimus preferentially supports Tregs generation as Teffectors (Teffs) are mTOR-dependent whereas *in vitro* or *in vivo* induced peripheral Tregs and FoxP3 expression are favored by mTORC1 complex inhibition by sirolimus (61). Antimetabolites predominantly methotrexate, a folic acid antagonist and mycophenolate mofetil, an inhibitor of the *de novo* purine metabolism are being used with other immunosuppressants in allo-HSCT patients (56, 58, 59).

## Inhibiting Co-stimulatory Signals

It is well established that the fine-tuned balance between co-stimulation and inhibitory signals dictates immune responses (104, 105). Numerous co-stimulatory and co-inhibitory molecules have been identified and targeted to prevent and reduce various inflammatory diseases including GVHD. Preclinical studies of co-stimulatory and co-inhibitor pathway blockade for GVHD prevention have been comprehensively reviewed recently; the reader is referred to (22). Here we will focus on clinical trial results to prevent GVHD using CTLA4-Ig to block B7/CD28 co-stimulation.

The two-signal model of T cell activation required that both antigen and secondary stimuli are essential for optimal T cell activation (106, 107). The co-stimulatory CD28, identified as a receptor for B7-1 (CD80) ligand and B7-2 (CD86), is constitutively expressed on T cells (108–112). CD28 signals support T cell growth and survival. The co-inhibitory receptor CTLA-4, which also binds to B7-1 and B7-2, serves to temper T cell responses in part by down-regulating CD28 expression.

Linsley and coworkers developed CTLA-4 Ig, consisting of the extracellular CTLA-4 domain, and an immunoglobulin Fc fragment fusion partner to prolong its half-life, as a therapeutic agent that binds and sequesters B7 ligands from CD28 engagement (22). Earlier studies from the 1990s, including from our group, reported the efficacy of CTLA-4 Ig in the prevention of autoimmunity, solid organ allograft rejections and GVHD lethality (113–115) in murine models. The results from these studies laid the foundation for the first clinical trial (NCT01012492) in GVHD using abatacept (humanized CTLA4-Ig fusion protein) that showed a reduced GVHD incidence (62). Phase II studies (NCT01743131) testing the efficacy of abatacept against standard GVHD prophylaxis has been completed for in 7–8/8 HLA matched related or unrelated donor transplants.

The immunomodulatory effect of abatacept was associated with increased expression of PD-1 on T cells of the clinical responders. The role of the PD-1 pathway in inducing immune tolerance and controlling acute GVHD has been well established (24, 116). Although the beneficial effect of abatacept depends on blocking CD28 co-stimulation, it can also interfere with the endogenous CTLA-4 co-inhibition pathway and can lead to unwanted immune responses (117). The advent of fusion proteins or antibodies that block only the CD28 pathway without interfering with CTLA-4 may have an edge over abatacept due to their specificity. Belatacept, a 2 amino acid derivative of abatacept, was developed as a selective co-stimulatory pathway blocker, that has favorable results in renal transplant rejection compared to cyclosporine prophylaxis (118). A CD28 antagonistic antibody, FR104, has been tested in various pre-clinical models (119–121). More recently, in a non-human primate (NHP) GVHD model, compared to CTLA4-Ig or CTLA4-Ig/sirolimus prophylaxis, FR104 or combined FR104/sirolimus prophylaxis delayed the onset of GVHD by controlling T cell activation and proliferation (122). However, there were non-GVHD-related deaths in the FR104/sirolimus-treated NHP due to sepsis. Detailed immunological analysis revealed that T cells from those primates failed to produce IFN-γ. The results from this study still highlight FR104/sirolimus combination as a promising therapy to treat GVHD in human patients due to better infection control compared to NHP.

## Impairing Activating and Inflammatory Cytokine Signals Driven GVHD Injury

### Immune Activating Cytokines Contributing to GVHD

JAKs are intracellular tyrosine kinases and act as downstream of cytokines, growth factors and hormone signaling. The JAK family members comprises JAK1, JAK2, JAK3, and TYK2 (123). JAK signaling supports the development, proliferation, and activation of T- and B- cells, DCs, macrophages, and neutrophils, all implicated in GVHD pathogenesis.

Ruxolitinib, a selective inhibitor of JAK1 and JAK2 reduced GVHD, associated with decreased proinflammatory cytokine production, Th1 differentiation and increased Tregs proportions (124, 125). Although ruxolitinib has been primarily reported as a treatment for steroid refractory or resistant GVHD (63), a recent study in myelofibrosis patient reported that ruxolitinib, given during peritransplant period, can reduce GVHD (64). Overall, 1 out of 12 patients developed severe (grade III) GVHD without major events during conditioning. However, CMV reactivation was seen in 4 of 6 CMV positive patients and 2 had cytopenias requiring ruxolitinib discontinuation (64). In other studies (126), baricitinib, a best-in-class Jak1/2 inhibitor, blunted IFNγR and IL6R signaling, resulting in complete protection from GVHD lethality as well as the reversal of active GVHD prevents GVHD with 100% survival, and reverses ongoing GVHD with dramatically increased Tregs along with decreased Th1 and Th2 differentiation, MHC class II and B7 ligand expression on APCs (126). Pacritinib is a potent JAK2 inhibitor that can reduce GVHD by sparing iTregs and polarizing T cells toward Th2 differentiation (65). A phase I/II trial (NCT02891603) combining pacritinib with standard immune suppression to prevent GVHD is currently being investigated.

Tofacitinib, a first generation JAK1/JAK3 inhibitor, reduced murine GVHD lethality (127). Antibodies directed to the IL2R common gamma chain that signals via JAK3 and STAT5 reduce proinflammatory cytokine production, CD8+ T cell granzyme B expression and severe GVHD lethality (128). Indeed JAK3 knockout T cells were unable to cause GVHD mortality in sublethally irradiated MHC class II disparate recipients. Pharmacological JAK3 inhibition with WHI-P131 given as prophylaxis ameliorated GVHD severity with a prolonged survival when compared to control mice (129). As many of these reagents are in the clinic including for GVHD prevention they may become part of an *in vivo* approach to achieve tolerance.

### Alpha-1-antitrypsin to Reduce Pro-inflammatory Responses Post-transplant

Alpha-1-antitrypsin (AAT) is an acute phase secretory protein and a serine proteinase inhibitor, elevated during inflammation due to its predominant synthesis in hepatocytes (130, 131). Numerous lines of evidence demonstrated the anti-inflammatory properties of AAT. Studies have shown that the deficiency of AAT aggravated the severity of inflammatory disease, whereas addition of AAT to LPS-stimulated monocytes or mononuclear cells inhibited the release of pro-inflammatory cytokines (132–134). In GVHD patients, there was a negative correlation between AAT levels in donor plasma and occurrence of GVHD (135). Indeed, AAT treatment attenuated the lethality of GVHD in pre-clinical murine models by both increasing IL-10 levels and numbers of Tregs, and reducing the levels of pro-inflammatory cytokines such as IL1-β, TNF-α, and IL-6 (66, 135, 136). This tolerogenic effect of AAT, which induced Tregs expansion, was mediated by an increase in the numbers of CD8^+^ CD205^+^ DCs (135). AAT strongly inhibits neutrophil elastase and that may also contribute to reduced GVHD lethality due to the pathogenic role of neutrophils in GVHD (38). In clinical trials (NCT01523821 and NCT01700036), AAT treatment increased the proportion of Tregs and reduced GVHD manifestations (67), while decreasing numbers of CD8^+^ TM cells (68) in steroid refractory (SR) GVHD patients without clinical toxicity.

### Regulating Histone Deacetylase

Histone acetylation epigenetically regulates cell function by modulating gene expression. Acetylation is often associated with transcription activation, while deacetylation is associated with repression. The interplay between histone acetyltransferases (HATs) and histone deacetylases (HDAC) influences histone acetylation to impact numerous cellular functions, including cell differentiation, and apoptosis (56). HDAC inhibitors (HDACi) function an anti-inflammatory agents in autoimmune and inflammatory disorders (137). HDACi, namely vorinostat (SAHA), romidepsin (Istodax) and panobinostat (LBH589), are FDA-approved agents to treat cancers. HDACi treatment ameliorated murine GVHD through upregulation of indoleamine 2,3-dioxygenase (IDO) in DCs, in a STAT-3-dependent pathway (138, 139). Trytophan depletion and/or the generation of tryptophan catabolites has proven to be immune suppressive for murine GVHD (140, 141) as discussed in detail below. A completed phase I/II clinical trial (NCT00810602) of vorinostat with standard GVHD prophylaxis in patients who received matched related donor allo-HSCT reported reduced GVHD with lower levels of plasma IL-1β, TNF-α, IL-6, and IL-8 (69–71). Furthermore, HDACi treatment increased Treg cell numbers and enhanced their function in those patients (71). Extending this treatment to unrelated donor HCT (NCT01790568) also showed vorinostat to be result in a low rate of GVHD (70).

## Blocking T Cell Chemokine Receptor Directed Migration Into GVHD Organs

Chemokine receptors control the trafficking of T cells into tissues, where they may be primed, re-stimulated in the case of memory T cells, or cause cytolysis and tissue destruction. Chemokines produced by tissues injured by the conditioning regimen or GVHD itself may result in the elaboration of chemokines that direct the recruitment of specific innate and adaptive immune cells. Chemokine and chemokine receptor interactions that can influence GVHD pathogenesis have been reviewed (142). For example, during tissue damage, the up-regulation of CCR5 directs lymphocyte homing to the inflamed intestine and liver tissues (143–146). In mouse GVHD models, the efficacy of CCR5 blockade was dependent upon the degree of conditioning regimen injury. Whereas, anti-CCR5 mAb prevented T cell homing to Peyer's patches in the absence of conditioning (146), GVHD was accelerated with lethal radiation conditioning due to increased T cell expansion, IFN-γ and TNF-α production, and infiltration into the liver and lung (144, 146). In patients, reduced CCR5 expression correlated with lower GVHD (147, 148). Short-term addition of CCR5 antagonist, maraviroc added to standard GVHD prophylaxis resulted in reduced GI and liver GVHD in allo-HSCT patients given reduced intensity conditioning (72). Compared to this short-term treatment of 1 month (72), the extended course of maraviroc (3 months) was also safe and resulted in a significantly improved survival and higher GVHD-free (73). The relationship between conditioning regimen intensity and efficacy of CCR5 antagonism in allo-HSCT patients is unknown and warrants investigation.

Studies have demonstrated that the expression of gut-homing molecules, including α4β7-integrin and chemokine receptor CCR9, by T cells is required for homing to the intestines. GI injury due to conditioning is a key trigger for GVHD pathogenesis and results in the homing of donor T cells to the injured GI tract. Natalizumab is a potential drug of interest to mitigate GI GVHD due to its selective inhibition against α4 integrins of α4β7. Natalizumab and vedolizumab, a specific anti-α4β7 integrin monoclonal antibody, have been used in for GVHD treatment but not prevention, which have distinct cellular infiltrates and pathophysiologies (149, 150). Homing receptor blockade may potentiate tolerance induction in allo-HSCT as GVHD by precluding immune cell recruitment into GVHD organs and amplification of tissue injury.

## Regulating GVHD by Exploiting Cellular Metabolism Mechanisms

### Intrinsic T Cell Metabolic Energy Sources Required for GVHD

One way to tailor immune tolerance is to change the metabolic fitness. Immune cells require considerable bioenergy to generate and sustain immune responses against pathogens, allografts, and tumor cells. To accomplish these effector responses, immune cells utilize multiple metabolic pathways. The major metabolic pathways involved in cellular growth and proliferation are tricarboxylic acid (TCA) cycle, glycolysis, amino acids, pentose phosphate, fatty acid synthesis and oxidation (151, 152). Despite their diverse end products, these pathways are interdependent as biosynthesis of one pathway depends on the intermediate products of other pathways.

The TCA cycle takes place in the mitochondria to generate energy through oxidation of acetyl CoA, which is derived from sources such as glucose, fatty acids (FA) and glutamine (151, 152). The end products of the TCA cycle, namely NADH and FADH2 contribute electrons into the electron transport chain (ETC). The ETC is involved in highly efficient ATP generation by supporting oxidative phosphorylation (OXPHOS). Metabolically quiescent cells, like naive T cells, generate energy via OXPHOS by fueling TCA cycle with the available nutrients. However, upon cognate antigen encounter, T cells undergo a metabolic switch from OXPHOS to glycolysis to meet their energy needs (151–153). In glycolysis, extracellular glucose enters the cell through glucose transporters followed by the sequential conversion of glucose to pyruvate and other products by different enzymes. The availability of oxygen in the cell influences the fate of pyruvate. In the case of hypoxia, pyruvate is converted to lactate and NAD+. However, in normoxia, pyruvate is oxidized through the TCA cycle. Glycolysis plays a crucial role in cellular metabolism by providing precursors to other metabolic pathways. For example, cytoplasmic acetyl-CoA, a metabolite of glycolysis, promotes lipid synthesis by generating cholesterol and fatty acids. In a preclinical model, donor T cells shown to increase oxidative phosphorylation in both syngeneic and allogeneic recipients (153, 154). Glycolytic activity was only higher in donor T cells of allogeneic recipients than those of acute GVHD controls or syngeneic BMT recipients, indicating that Teffector cells causing GVHD are more dependent upon glycolysis (154–156). Pharmacological inhibition of mTORC1 or a phosphofructosekinase-2 isoform PFKB3 reduced GVHD lethality (154). Moreover, mice given T cell deficient in the glucose transport glut-1 were unable to induce GVHD (157).

Apart from glycolysis, glucose can also be metabolized via the pentose phosphate pathway (PPP) and glycogen synthesis (151). PPP is comprised of oxidative and non-oxidative branches. The oxidative branch of PPP maintains the cellular redox environment by generating reducing equivalents of NADPH. Whereas, the non-oxidative branch supports cell proliferation by generating required nucleotide and amino acid precursors (151). During GVHD, PPP activity of alloreactive T cells was increased (154).

Fatty acid oxidation (FAO) generates energy by converting FA to acetyl CoA, which enters into the TCA cycle (151, 153). In addition, FAO supports the ETC production of ATP by generating NADH and FADH2. Short and medium chain FA passively diffuse into the mitochondria, whereas the carnitine palmitoyl transferase (CPT) system regulates long chain FA (C14 to C18) metabolism (158). Discordant results have been reported about the activity of FAO in alloreactive T cells with some studies reporting increased FAO (155, 159), while a recent one demonstrated diminished FAO (154). FA synthesis plays a crucial role in sustaining T cell proliferation by generating lipids through utilization of products derived from other metabolic pathways (151, 153). Lipid synthesis is regulated by enzymes such as acetyl-CoA carboxylases (ACC 1 and 2) and fatty acid synthase (151, 153, 154). Deficiency of ACC1 in donor T cells ameliorated GVHD due to impaired *de novo* FA synthesis (160). Sphingolipids are major components of eukaryotic cell membranes and play a crucial role in cellular survival, proliferation, differentiation and growth arrest. A recent study reported that ceramide, a metabolite of sphingolipids, modulate GVHD lethality (161). Targeting ceramide synthase 6, a ceramide biosynthetic enzyme, by either genetic deletion in donor T cells or pharmacological inhibition ameliorated GVHD due to reduced donor T cell proliferation and Th1 differentiation (161).

Glutamine, a key amino acid and readily available resource in serum, is required for T cell activation (5). Glutamine is involved in nucleotide synthesis and its metabolite glutamate also fosters the TCA cycle, glutathione and amino synthesis (151, 153). In allo-HSCT, donor T cells upregulated glutamine transport channels namely glutaminase 2, phosphoribosyl pyrophosphate amidotransferase, and glutamine-fructose-6-phosphate transaminase to increase the uptake of glutamine (25, 154). Furthermore, only donor T cells from allo-HSCT had increased levels of glutamate products aspartate and ornithine, which indicate that donor T cells can restore the exhausted intermediates of TCA cycle by increasing glutaminolysis (153, 154). Based on these studies, strategies inhibiting glycolysis, fatty acid oxidation, oxidative phosphorylation, or glutaminolysis may be an area of great potential to control GVHD (5).

### Extrinsic Regulation of Cellular Metabolism in GVHD

A defense mechanism against GVHD lethality can be conferred by essential amino acid depletion results in a state of metabolic starvation. For example, high host tissue expression of IDO that catabolizes and hence depletes L-tryptophan was critical to reduce colonic GVHD (140). Donor T cell-derived IFN-γ upregulated the expression of IDO in colonic epithelial cells which in turn, diminished T cell proliferation and inflammation (141). Similarly, IDO expression was upregulated in the duodenal epithelial cells of GVHD patients and may be involved in the control of GI GVHD (162). The metabolic products of tryptophan catabolism has been shown to be immune suppressive. Whereas, combined administration of three tryptophan metabolites suppressed GVHD, kynurenines given in this way did not appear to be tolerogenic since GVHD was controlled only during the continuous administration period (141). In other studies, arginine depletion by myeloid-derived suppressor cell production of arginase I or infusion of pegylated L-arginase I was shown to reduce the vigor of the GVHD lethality response (163).

Mammalian hosts harbor a large number and a wide variety of commensal bacteria on surfaces of the body, especially in the GI tract. Commensal bacterial density in the GI ranges from 10^11^ to 10^14^ per gram of luminal content (164). The interaction between GI commensals and host immune cells plays a critical role in the development of the immune system and the maintenance of intestinal immune homeostasis. For example, germ-free mice have impaired immune systems with smaller Peyer's patches, lower numbers of IgA-producing plasma cells and lower numbers of CD8+ intraepithelial cells (165, 166). Dysregulation of GI microbiota has been associated with various inflammatory diseases (167–169).

In addition to metabolizing host dietary components, microbes produce their own metabolites that can have substantial immune system effects (170–173). In GVHD mice (174, 175) and patients (176–178), the diversity of the intestinal microbiota is significantly altered, which can be associated with the lethality of the disease. For example, butyrate, a short chain fatty acid microbial metabolite is an HDACi serves as the main energy source of intestinal epithelial cells (IECs) (179, 180). In a mouse model of GVHD, the reduction in intestinal butyrate resulted in decreased histone acetylation within CD326+ IECs (181). Administration of exogenous butyrate mitigated GVHD by increasing both anti-apoptotic and junctional proteins of IECs. This beneficial effect was not found to be mediated by donor Tregs, however the role of host Tregs in this model remains to be explored. Similarly, intragastric gavage of 17 rationally-selected strains of high butyrate–producing *Clostridia* also reduced GVHD and improved survival (181). A clinical trial (NCT02763033), which aims to increase butyrate levels in the intestines using dietary supplements containing potato-based resistant starch, is ongoing. Overall, these results demonstrate that HDACi can mitigate GVHD lethality.

In addition to butyrate, a recent study (182) reported that allo-HSCT conditioning regimens reduced indole or indole derivatives due to altered intestinal microbiome. Importantly, either supplementation with exogenous indole derivative or colonization of bacteria that can deliver indole metabolites into intestines of allogeneic murine recipients ameliorated GVHD lethality with reduced mucosal damage and pro-inflammatory cytokines (182). Beyond GVHD amelioration, recipient-specific tolerance was developed in donor T cells of recipients that were administered with the tryptophan metabolite and indole derivative, indole-3-carboxaldehyde, found in foods such as collard greens and broccoli.

## Tissue Tolerance Mechanisms

It is a known fact that the survival of host against infections depends on the capacity of host's immune system. Recently, the role of parenchymal tissues on reducing disease severity and protecting from immunopathology has been gaining attention as tissues can modulate immune responses (183, 184). In non-infectious disease settings like GVHD, tissue tolerance is defined as an intrinsic and protective mechanism of parenchymal tissue to ameliorate GVHD against ongoing alloimmune responses. Studies from our laboratory demonstrated that the expression of the co-inhibitory molecule by parenchymal tissues promoted tolerance and reduced the lethality of GVHD. Experimental evidence has demonstrated the increased expression of co-inhibitory molecules such as programmed death-1 ligands and B7-H3 on T cells in GVHD targeted tissues (27, 156). The absence of these molecules accelerated GVHD lethality due to augmented T cell effector responses. Thus, co-inhibitory pathways induced during alloresponses serve to dampen alloreactive donor T cell responses and hence GVHD.

## Reparative Processes

Emerging data suggest that tissue tolerance can also be mediated through the regeneration of damaged tissues. In a recent study, administration of the Wnt-agonist R-spondin1 mitigated GVHD by protecting intestinal stem cells (ISC) and facilitating repair of the intestinal epithelium (185). In line with this finding, IL-22, which has been shown to activate ISC, enhanced intestinal epithelial regeneration and ameliorated GVHD (186). A phase I/II clinical trial (NCT02406651) is currently investigating the safety and efficacy of use of IL-22 in combination with corticosteroids for the treatment of patients with newly diagnosed GI GVHD.

Tregs and innate lymphoid cells 2 (ILC2) aid tissue repair by secreting amphiregulin, an epidermal growth factor that promotes tissue repair under inflammatory conditions (187). Non-lymphoid cells, in particular, mesenchymal stem cells (MSCs), have also been shown to facilitate tissue repair by polarizing tissue macrophages to the anti-inflammatory phenotype (188). These macrophages help repair tissues through enhanced fibroblastic proliferation and also reduce donor T cell proliferation and so limit GVHD. Furthermore, MSCs promote tissue repair by increasing the proliferation of ILC3 and their subsequent IL-22 production (189). Overall, strategies harnessing tissue tolerance represent a novel and expanding area of research in GVHD.

## Cellular Therapies

Infusions of tolerogenic cells are one of the most attractive strategies to achieve long-term immune tolerance in clinical studies due to the long-term persistence of those cells. There are numerous immunoregulatory cells that have been used to induce transplantation tolerance in clinical models, but herein we will focus on Tregs, invariant natural killer T (iNKT) cells (see also Dominik Schneidawind's chapter) and MSCs.

### Mixed Hematopoietic Chimerism and Tolerance Induction

Mixed hematopoietic chimerism also has been shown to be to facilitate kidney and liver solid organ graft acceptance in mice and humans (190–193), with high levels causing central deletional tolerance albeit at the risk of GVHD and transient chimerism allowing for peripheral tolerance that begins with Treg mediated mechanisms and transitions into peripheral tolerance likely including deletion of donor alloreactive T cells (74, 194–196). While transient T cell chimerism in hematological malignancy patients can decrease GVHD (75), mixed donor T cell chimerism present on day 90 in allo-HSCT patients receiving a reduced intensity conditioning regimen did not preclude GVHD generation; however the incidence was significantly lower than those with full donor T cell chimerism (35 vs. 61%), providing a platform upon which to tolerance induction may be more likely to be achieved (76).

### T Regulatory Cell Infusion for Tolerance Induction

Tregs play a crucial role in maintaining immune homeostasis and tolerance by preventing autoimmunity and immunopathology. Tregs may be derived from the thymus (thymic-derived or natural Tregs (tTregs or nTregs), peripherally derived Tregs (pTregs), and *in vitro* induced Tregs (iTregs) (197). In this review, we will focus on both basic and clinical studies using different subsets of Tregs for the prevention of GVHD and discuss their limitations.

### Thymic-Derived Tregs

Phenotypic features of tTregs include the constitutive expression of CD25, the high- affinity IL-2 receptor, CTLA-4, and Forkhead box P3 (Foxp3), a lineage transcription factor. Adoptive transfer of tTregs has been demonstrated to control allograft rejection and GVHD by limiting alloimmune responses (198–200). Preclinical studies have shown a high efficacy of Treg infusion and GVHD prevention (201–203). In allo-HSCT patients, there was an inverse correlation between Treg frequency and risk of acute GVHD (204).

Translation to the clinic proved challenging due to the low frequency of tTregs (typically 2–3% of CD4+ T cells) in the peripheral blood (205). The phenotypic profile of human tTregs was not as readily demarcated in peripheral blood as in the spleen and lymph nodes of mice. Moreover, compared to non-Treg T cells, Tregs were found to be hyporesponsive resulting in poor expansile properties and a preponderance of contaminating non-Tregs even when the latter represented a minor proportion of input cells. The first acute GVHD prevention clinical studies were reported by two groups (77, 78). In our study (77), umbilical cord blood cells were used as a source of tTregs (NCT00602693). Advantages included ease of tTreg isolation as a result of higher frequency of CD4+CD25bright cells and reduced likelihood of CD25+ Teffs contamination due to fetal microenvironment that minimizes external antigen exposure. *Ex vivo* expansion permitted tTreg activation, maximizing expansion and suppressor function. GVHD was reduced but not eliminated at Treg:Teff ratios of ≤1:6 in patients receiving cyclosporine A or sirolimus and mycophenolate. In the second study by our group, tTreg expansion was dramatically increased by restimulating tTregs with cell-based artificial antigen presenting cells and when given to patients receiving sirolimus and mycophenolate mofetil, GVHD was virtually eliminated (79). In the study by Martelli and coworkers (78), tTregs were freshly isolated from peripheral blood and allowed to become activated and expanded *in vivo* prior to the infusion of haploidentical T cells and in the absence of post-transplant immune suppression. GVHD was very low considering the high T cell dose given. Since tTregs could not be detected in peripheral blood beyond ~2 weeks post-transplant, these studies suggest that tTregs have tolerized the donor T cell graft. In other studies, antigen-specific tTregs have been generated and expanded *in vitro* in rodents (206) and are being tested in the clinic for GVHD prevention.

Tregs rely on IL-2 for their generation, proliferation, lineage stability and survival; however, they are poor producers of IL-2 (207). In patients, ultra-low dose IL-2 given as GVHD prophylaxis days 7–30 resulted in Treg expansion *in vivo* and no instances of GVHD in 16 pediatric allo-HSCT recipients (208). In patients with cGVHD, low dose IL-2 administration ameliorated cGVHD lethality by preferentially allowing *in vivo* Treg expansion, increasing the Treg:Teff ratio and thus favoring tolerance (209, 210). Since both Tregs and activated Teffs respond to IL-2, it is currently unknown whether these studies can be extrapolated to the higher risk adult population, which may be benefitted by more selective Treg expansion approaches. For example, two recent studies employed novel approach to selectively target Treg expansion. In one of the studies (211), investigators engineered IL-2 cytokine-receptor orthogonal pairs that interact with one another but not with their natural cytokine and receptor counterparts. Introduction of a mutated IL-2Rβ into T cells that preferentially binds orthogonal but not natural IL-2 enabled the selective cellular targeting of engineered T cells *in vitro* and *in vivo*, with limited off-target effects and negligible toxicity, suggesting a clinical strategy to selectively target Tregs *in vivo* in patients. In a different study, the same group employed complexes of human IL-2 with a unique conformational structure that stabilized IL-2 and promoted preferential STAT5 phosphorylation and Treg expansion (212).

In a non-IL-2 based approach, investigators have used reagents that stimulate death receptor 3 (DR3, TNFRSF25), a member of the tumor necrosis factor (TNF) receptor superfamily primarily expressed on Tregs, lymphoid tissue inducer cells, and NKT cells (213). The natural ligand of DR3, TL1a, is expressed on endothelial cells and APCs (213). An agonistic αDR3 mAb significantly expanded Tregs *in vivo* and prevented the development of allergic lung inflammation (214) and cardiac allograft survival by increasing the proportion of Tregs (215). Treating donors with αDR3 preferentially allowed Tregs expansion with reduced Tcon activation and those donor T cells reduced GVHD (216). A key role of TNF binding to TNFR2 was discovered to be critical to Treg control of GVHD (217, 218). Collectively, strategies to increase the tTreg/Teffs *in vivo* represent a promising therapeutic option to reduce GVHD and remain an active area of research.

#### Inducible Tregs (iTregs)

Although tTreg cellular therapy has great potential in controlling GVHD, higher doses of Tregs are required and it has been challenging to achieve uniform and robust tTreg expansion in clinics. Generation of iTregs is an alternative strategy to overcome the obstacle of limited nTreg cell numbers. Previous studies have established the potency of iTregs in controlling various autoimmune disorders (219, 220). In an experimental GVHD study, antigen-specific iTregs were generated and they were able to reduce GVHD by inhibiting the activation, proliferation and migration of donor T cells (221). The methylation status of the Treg-specific demethylated region (TSDR) of the Foxp3 promoter determines the stability of Tregs by maintaining the stable expression of Foxp3 (222, 223). Unlike tTregs, iTregs are completely methylated at the TSDR and tend to be unstable in GVHD mice (80, 224). Hence, studies have attempted to use various agents such as rapamycin, retinoic acid, and IL-6 blockade to induce and maintain iTregs (224). However, only the usage of sirolimus both *in vitro* and *in vivo* was shown to improve CD4+ iTreg stability in a mouse model of GVHD (80). Given the role of iTregs in controlling GVHD, there is an ongoing phase-I trial (NCT01634217) initiated by our institute to test the safety of CD4+ iTregs, generated using sirolimus, TGF-β, and IL-2, when given as GVHD prophylaxis to matched sibling donors along with CNI and mycophenolate thereby reducing the inflammatory environment. Intriguingly, CD4+ tTregs and iTregs were shown to be synergistic in controlling colitis in mice (81). A previous study reported that CD8+ iTregs can be induced by activating CD8+ CD25- T cells with allogeneic CD11c+ DCs, IL-2, TGF-β and retinoic acid. Although CD8+ iTregs expressed higher levels of suppressive molecules like CD39^+^CD73^+^, CTLA-4, and granzyme than CD4+ iTregs, there was no difference observed between their *in vitro* suppressive functions (82). In contrast to their *in vitro* suppressive functions, CD8+ iTregs are less potent than CD4+ iTregs in controlling GVHD due to their pro-apoptotic phenotype and thus reduced survival but are more effective in eliminating leukemia cells (82, 225). Intriguingly, CD8+ iTreg expression of FoxP3 can be stabilized by JAK2 targeting (226).

#### Type 1 T Regulatory (Tr1) Cells

Type 1 T regulatory (Tr1) cells are a distinct pTreg subset discovered by Bacchetta and Roncarolo and colleagues in severe combined immune deficiency patients who did not develop GVHD but had anti-host reactive T cell clones that produced high IL-10 and low IL-2 protein (227, 228). Tr1 cells lack constitutive expression of Foxp3, and have been shown to exert immune tolerance mainly via production of cytokines such as IL-10 and TGF-β (229, 230) that can inhibit murine GVHD lethality (228).

Using novel transgenic mice, Hill's group recently reported that Tr1 cells are the dominant immunoregulatory cells after allo-HSCT due to defective tTreg homeostasis (231). Infusion of Tr1 cells reduced GVHD, while Tr1 deficiency aggravated GVHD lethality. Murine and human Tr1 cells are typically generated by alloantigenic stimulator cell exposure in the presence of high IL-10 (83, 228, 229, 232). As with antigen-specific tTregs, Tr1 cells may have a reduced capacity for global immunosuppression due to their allospecificity. A recently completed phase-I trial demonstrated the feasibility of host-specific donor Tr1 therapy in GVHD patients. Infusions of Tr1 cells reduced GVHD, enhanced immune reconstitution and promoted tolerance induction (84). There are ongoing clinical trials testing the efficacy of Tr1 cell therapy in controlling autoimmunity and other inflammatory disorders (230).

### Invariant Natural Killer T (iNKT) Cells

iNKT cells are a rare lineage of immunomodulatory cells and they produce large quantities of anti-inflammatory cytokines such as IL-4 and IL-10 (85). Numerous lines of evidence have highlighted the potency of iNKT cells in promoting immune tolerance in GVHD (233). Studies from the early 2000s demonstrated that a combined regimen of fractionated total hematopoietic irradiation and depletion with anti-T cell antibodies reduced GVHD in rodent models (234, 235). The protective effect against GVHD was meditated by the expansion of host immunoregulatory iNKT cells, which secreted IL-4 and supported donor Treg proliferation (234–236). Pharmacological approaches to expand iNKT cells, using a synthetic iNKT TCR ligand, α-galactosylceramide (alphaGalCer), also attenuated GVHD (237). An important consideration in these studies was the usage of reduced conditioning regimens that may help in the survival of host iNKT cells and their expansion. However, using lethally irradiated GVHD mouse models, Negrin's group demonstrated that the lethality of GVHD could be mitigated by adoptive transfer of low numbers of recipient-type, donor-type, or third party iNKT cells (238–240). These studies shed light on the role of iNKT cells in expanding both donor Tregs and myeloid derived suppressor cells (MDSCs). Interestingly, the protective effects of iNKT cell and donor Treg expansions were dependent on MDSCs and thus, crosstalk between these distinct cell populations promoted immune tolerance in GVHD settings. Results from these experimental models led to the initiation of a phase-II trial (NCT01379209) in GVHD patients. This clinical trial used a single dose of RGI-2001, the liposomal formulation of α-GalCer to expand iNKT cells. While there was reduced GVHD and increased expansion of Tregs observed in some patients, iNKT cells were very low in number and difficult to detect in the peripheral blood (241). Clinical studies testing infusions of iNKT cells hold promise to control GVHD.

### Mesenchymal Stem Cells (MSCs)

Therapeutic infusions of MSCs are one of the leading options to treat GVHD. Although MSCs are rare non-hematopoietic cells in bone marrow, these cells are easy to isolate and can be expanded rapidly *in vitro* due to their multipotent and self-renewable properties (205). Immunomodulatory effects of MSCs in attenuating GVHD are mediated by secretion of cytokines (IL-6, TGF-β), soluble receptors (PDL-1, PDL-2) and effector molecules (nitric oxide, PGE2). MSCs also downregulate a wide range of chemokine (CCL1, CCL3, CCL8, CCL17, CCL22) expressions on donor T cells to limit T cell effector migration into target tissues (86, 205). The suppressive capacity of MSCs is enhanced by IFN-γ produced during GVHD, which up-regulates PDL-1 and IDO expression on MSCs to control T cell activation (205, 242). In other studies, high host anti-donor cytotoxic T lymphocyte (CTL) activity serves to eliminate donor MSCs and at the same time induce IDO and immune suppression by perforin-dependent host CTL mediated donor MSC apoptosis (243). Additionally, MSCs participate in the reparative process of tissue by promoting angiogenesis, regeneration, and remodeling (205). These properties have led to multiple clinical trials (NCT03158896, NCT00284986, NCT00361049, NCT00366145, NCT02336230) exploring the use of MSC infusion as an adjunctive strategy for GVHD prevention (87).

## Concluding Remarks

Recently, there have been significant advances in the field of allo-HSCT to treat GVHD. Early phase studies involving AAT, HDACi and co-stimulation blockade have shown promising results, although randomized clinical trials and longer follow-up will be required to validate these existing results. Adoptive cellular therapies are powerful strategies to achieve peripheral tolerance swiftly in allo-HSCT recipients by blunting the inflammatory component of GVHD. Clinical trials using tTregs have reported promising results, but the long-term effects of Tregs on immune responses against infections and tumors have yet to be determined. To reduce non-specific immunosuppression and increase potency of antigen-specific suppression, generation of antigen-specific Tregs by a variety of approaches including engineering Tregs using chimeric antigen receptors (CAR) or designated T-cell receptors reactive against antigens present in GVHD organs may be an attractive approach. The first clinical trial evaluating CAR Treg therapy in the prevention of organ transplant rejection is expected to start by next year. Gene augmentation and gene editing techniques may be employed to direct Tregs to particular GVHD organs such as the gut or to increase Treg stability under inflammatory conditions. Renewed efforts are required to gain insight into tolerance induction in allo-HSCT and to develop safe and effective strategies to combat GVHD.

## Author Contributions

GT wrote the manuscript. BB assisted in the writing and editing of the manuscript.

### Conflict of Interest Statement

The authors declare that the research was conducted in the absence of any commercial or financial relationships that could be construed as a potential conflict of interest.
